# Suppression of HopZ Effector-Triggered Plant Immunity in a Natural Pathosystem

**DOI:** 10.3389/fpls.2018.00977

**Published:** 2018-08-14

**Authors:** José S. Rufián, Ainhoa Lucía, Javier Rueda-Blanco, Adela Zumaquero, Carlos M. Guevara, Inmaculada Ortiz-Martín, Gonzalo Ruiz-Aldea, Alberto P. Macho, Carmen R. Beuzón, Javier Ruiz-Albert

**Affiliations:** ^1^Departamento Biología Celular, Genética y Fisiología, Instituto de Hortofruticultura Subtropical y Mediterránea, Universidad de Málaga-Consejo Superior de Investigaciones Científicas, Málaga, Spain; ^2^Shanghai Center for Plant Stress Biology, CAS Center for Excellence in Molecular Plant Sciences, Shanghai Institutes of Biological Sciences, Chinese Academy of Sciences, Shanghai, China

**Keywords:** Type III secretion system, effector, ETI, suppression, plant defense, *Pseudomonas syringae*, HopZ1, HopZ3

## Abstract

Many type III-secreted effectors suppress plant defenses, but can also activate effector-triggered immunity (ETI) in resistant backgrounds. ETI suppression has been shown for a number of type III effectors (T3Es) and ETI-suppressing effectors are considered part of the arms race model for the co-evolution of bacterial virulence and plant defense. However, ETI suppression activities have been shown mostly between effectors not being naturally expressed within the same strain. Furthermore, evolution of effector families is rarely explained taking into account that selective pressure against ETI-triggering effectors may be compensated by ETI-suppressing effector(s) translocated by the same strain. The HopZ effector family is one of the most diverse, displaying a high rate of loss and gain of alleles, which reflects opposing selective pressures. HopZ effectors trigger defense responses in a variety of crops and some have been shown to suppress different plant defenses. Mutational changes in the sequence of ETI-triggering effectors have been proposed to result in the avoidance of detection by their respective hosts, in a process called pathoadaptation. We analyze how deleting or overexpressing HopZ1a and HopZ3 affects virulence of HopZ-encoding and non-encoding strains. We find that both effectors trigger immunity in their plant hosts only when delivered from heterologous strains, while immunity is suppressed when delivered from their native strains. We carried out screens aimed at identifying the determinant(s) suppressing HopZ1a-triggered and HopZ3-triggered immunity within their native strains, and identified several effectors displaying suppression of HopZ3-triggered immunity. We propose effector-mediated cross-suppression of ETI as an additional force driving evolution of the HopZ family.

## Introduction

Type III effectors (T3Es) are bacterial proteins translocated by complex and specialized molecular machines, type III secretion systems (T3SS), directly into the cytosol of eukaryotic cells, where they modify a variety of host cellular processes. The YopJ effector superfamily, named after the archetypal *Yersinia* effector, is formed by multiple members from both animal and plant pathogens and includes a group of effectors from *Pseudomonas syringae* known as HopZ effectors (Ma et al., 2006; Lewis et al., 2014). The HopZ family is in itself quite diverse, and is present in many *P. syringae* pathovars, with HopZ-carrying strains described to date including only one HopZ apiece (Ma et al., 2006; Baltrus et al., 2011; Üstün et al., 2014). The originally described HopZ T3Es included the HopZ1 allelic series, namely HopZ1a, HopZ1b, and HopZ1c, which seem to have evolved in *P. syringae*, along with HopZ2 and HopZ3 that are likely to have been acquired by horizontal gene transfer (Ma et al., 2006). Since then, additional members of the family have been described, such as HopZ4 or HopZ5, both likely acquired from *Xanthomonas* through horizontal gene transfer (Üstün et al., 2014; Jayaraman et al., 2017).

Several HopZ effectors display host defense suppression abilities. The first to be characterized, HopZ1a, suppresses several layers of the plant defense response, including pathogen-associated molecular pattern (PAMP)-triggered immunity (PTI) and effector-triggered immunity (ETI), as well as systemic acquired resistance (SAR) (Macho et al., 2009, 2010; Lewis et al., 2014; Rufián et al., 2015). To date, HopZ1a has been shown to function as an acetyltransferase (Lee A.H. et al., 2012; Jiang et al., 2013; Lewis et al., 2013; Ma et al., 2015; Rufián et al., 2015), and an assortment of host proteins have been proposed as targets of its virulence function (Zhou et al., 2011; Jiang et al., 2013). However, the exact nature of its relevant virulence target(s) within the plant is still under discussion. While very detailed, the characterization of HopZ1a virulence and avirulence activities has been performed either via heterologous expression from *P. syringae* strains that do not natively carry *hopZ* genes in *Arabidopsis*, or via *Agrobacterium*-mediated transient or stable expression in *Arabidopsis* and/or *Nicotiana benthamiana* (Lewis et al., 2010, 2014; Macho et al., 2010; Jiang et al., 2013). Thus, HopZ1a has been shown to suppress the ETI triggered by heterologous effectors AvrRpt2, AvrRps4, and AvrRpm1 in *Arabidopsis* (Macho et al., 2010). The fact that *P. syringae* strains natively carrying HopZ1a are poorly characterized and/or have been isolated from technically challenging host plants has probably hindered analysis in a native pathosystem (Ma et al., 2006).

In the case of HopZ3, heterologous expression has also been widely used in the characterization of its defense suppression ability, however, this has also been analyzed in the context of its native *P. syringae* strain B728a (hereafter *Psy* B728a), a fully sequenced model strain with a well-defined effector inventory (Vinatzer et al., 2006; Lee et al., 2015). HopZ3 has been shown to suppress the ETI triggered by several T3Es from the same effector repertoire, including HopAA1, AvrPto1, HopAE1, and HopM1, as determined by co-expression assays in *N. benthamiana*, a host plant for *Psy* B728a (Vinatzer et al., 2006), and also AvrB3 and AvrRpm1, as determined by co-expression assays in *Arabidopsis*, a non-host species (Vinatzer et al., 2006; Lee et al., 2015). In the latter case, HopZ3 achieves ETI suppression by acetylating a number of *Arabidopsis* defense proteins belonging to the RPM1 immune complex (Lee et al., 2015). Interestingly, HopZ3 can also interact with other effectors from the B728a repertoire that interfere with this defense hub (Lee et al., 2015).

As it often happens with T3Es, HopZ1a and HopZ3 suppress plant defenses as part of their virulence activity, but can also trigger ETI in resistant plants. HopZ1a-triggered ETI has been characterized through expression from non-native *P. syringae* strains and transgenic expression in *Arabidopsis*, where HopZ1a acetylation of the ZED1 pseudokinase, acting as a molecular decoy, leads to its detection by the ZAR1 resistance protein that triggers a defense response independent of salicylic acid or EDS1 (Lewis et al., 2010, 2013; Macho et al., 2010). Additionally, HopZ1a triggers ETI when transiently expressed in *N. benthamiana* and when expressed from heterologous strains in soybean or rice (Ma et al., 2006). HopZ3 does not trigger the hypersensitive response often associated to ETI when transiently expressed in *N. benthamiana* (except in those ectopically expressing the tomato R gene Pto) or *Arabidopsis* (Vinatzer et al., 2006; Lewis et al., 2008; Lee J. et al., 2012), but it does so in bean and tobacco, although in these cases the R-genes and overall molecular mechanisms involved have not been described yet.

The selective pressure exerted by the host immune system on T3Es can result in either the loss of the corresponding genes or in pathoadaptation, that is, mutational changes in the effector genes giving raise to new alleles that can avoid detection while retaining their virulence functions (Bartoli et al., 2016). In the case of the HopZ family, the allelic series comprising HopZ1a, HopZ1b, and HopZ1c has been proposed to originate through pathoadaptation, with HopZ1a being the closest to the ancestral allele (Ma et al., 2006). An alternative strategy also proposed for the HopZ family to avoid detection is the replacement of the detected alleles by homologs acquired by horizontal gene transfer, as suggested for HopZ2 or HopZ3 (Ma et al., 2006).

In this work, we analyze differences in virulence caused by the expression of HopZ1a and HopZ3 in their respective native strains in comparison with those caused by their expression on non-native strains. We show that both effectors trigger immunity in their plant hosts, but only when delivered from heterologous strains. We also show that immunity in these hosts is suppressed when HopZ1a or HopZ3 are expressed in their native backgrounds. We undertake two independent experimental approaches, suited to the particular characteristics of each of the native strains and ETI responses under study, to look for bacterial genes capable of suppressing HopZ1a-triggered or HopZ3-triggered immunity. We conclude that detectable suppression of HopZ1a-triggered immunity in the context of its native strain requires the combined action of more than one gene, perhaps encoding T3Es within the home repertoire. We also find several T3Es from *Psy* B728a that display suppressing activity on HopZ3-triggered immunity in bean, and could thus function as intra-repertoire suppressors of HopZ3-triggered immunity. Our data support the notion that the T3E repertoire of any given pathogen functions as a whole in determining the final outcome of a particular plant-pathogen interaction, and emphasize that the full evaluation of the biological relevance of any given T3E requires studies carried out in the context of the accompanying repertoire in its native strain.

## Materials and Methods

### Bacterial Strains and Growth Conditions

Bacterial strains used and generated in this work are listed in Supplementary Table S1. *Escherichia coli, P. syringae* and *Agrobacterium tumefaciens* strains were grown with aeration in lysogeny broth (LB) medium (Bertani, 1951) at 37°C (*E. coli*) or 28°C (*P. syringae* and *A. tumefaciens*). Antibiotics were used when appropriate at the following concentration: ampicillin (Amp), 100 μg/ml for *E. coli* and 300 μg/ml for *P. syringae*; kanamycin (Km), 50 μg/ml for *E. coli* and *A. tumefaciens* and 15 μg/ml for *P. syringae* derivative strains; rifampicin (Rf), 50 μg/ml for *A. tumefaciens;* gentamycin (Gm), 10 μg/ml; nitrofurantoin 50 μg/ml, and cycloheximide, 2 μg/ml.

### Plasmids and Cloning Procedures

All plasmids used in this work are listed in Supplementary Table S2. All PCRs were performed using Expand High Fidelity System (Roche, Germany) unless otherwise stated, and the primers used are listed in Supplementary Table S3. To generate the pMD1 derivatives, each corresponding open reading frame (ORF) was PCR-amplified using B728a DNA as template (for HopZ3 and HopAF1), or pEARLEYGATE103 (Earley et al., 2006) as template for green fluorescent protein (GFP), and cloned into pENTR/D (Invitrogen, Thermo Fisher Scientific, Waltham, MA, United States) into the *Not*I/*Asc*I sites to generate an entry clone, which in turn was subjected to a clonase reaction using a Gateway LR Clonase II Enzyme Mix (Invitrogen, Thermo Fisher Scientific, Waltham, MA, United States), and pMD1 as destination vector, to generate plasmids pMD1-Z3-3xFLAG, pMD1-AF1-3xFLAG, and pMD1-GFP-3xFLAG. To generate pJRU6, the ORF of HopZ1b was PCR-amplified using pUCP20tk::HopZ1b-HA (Zhou et al., 2009) as a template, and cloned into pAMEX in BamHI/XbaI sites. The ORF encoding HopZ3 and upstream ORF encoding its putative chaperone *schZ3* (Lewis et al., 2008) were PCR-amplified from genomic *Psy* B728a DNA and cloned into pAMEX in EcoRI/BamHI sites to obtain pCMG20 (Supplementary Table S2).

Vectors for allelic exchange were generated following the method described in Zumaquero et al. (2010). Separate PCR amplifications on *Psy* 7B40 or *Psy* B728a genomic DNA amplified 500 bps of the regions flanking the ORF to be deleted.

### Generation of Knockout Strains

Knockout strains were generated following a previously described method (Zumaquero et al., 2010). Briefly, allelic exchange vectors (Supplementary Table S2) were transformed by electroporation into *P. syringae*, then cultures were plated into LB plates supplemented with Km and determination of whether each clone was the result of plasmid integration (single recombination event) or allelic exchange (double recombination event) tested by replica plates onto LB and LB plates supplemented with Amp (300 μg/ml). Additional analysis of prospective clones included growth in liquid LB medium with 100 μg/ml of Amp (including 50 μg/ml of nitrofurantoin to avoid cross-contamination) and Southern blot analysis, using a 1,495 bp fragment of *nptII*-FRT as a probe to confirm that allelic exchange occurred at a single and correct position within the genome.

### Plant Material and Bacterial Inoculations

*Phaseolus vulgaris* cultivar Canadian Wonder plants were grown at 23°C, 95% humidity, with artificial light maintained for 16-h periods within the 24-h cycle. For *P. syringae* inoculum preparation, bacterial lawns were grown on LB plates for 48 h at 28°C, collected and suspended in 2 mL of 10 mM MgCl_2_. The OD_600_ was adjusted to 0.1 (approximately equivalent to 5 × 10^7^ colony forming units or cfu/mL) and serial dilutions made to reach the final inoculum concentration.

Plant inoculation by infiltration to be used for bacterial growth assays, or for monitoring disease symptoms, was carried out as follows: 10-day-old bean plants were inoculated with approximately 200 μl of a mixed bacterial suspension in 10 mM MgCl_2_, at the appropriate concentration, using a 1 ml syringe without needle.

### Competitive Index and Standard *in planta* Bacterial Replication Assays

CI assays in bean plants (*P. vulgaris* cv. Canadian wonder) were carried out as previously described (Macho et al., 2007). For inoculations by infiltration, 10-day-old bean plants, grown at 22–28°C with a photoperiod of 16/8 h light/dark cycle, were inoculated with 200 μl of a 5 × 10^4^ cfu/ml mixed bacterial suspension in 10 mM MgCl_2_, containing equal cfu of wild type and mutant or gene-expressing strain, using a 1 ml syringe without needle. Serial dilutions of the inoculum were plated onto LB agar and LB agar with the appropriate antibiotic to confirm by cfu counting the relative proportion between the co-inoculated strains, which should be close to 1. At 4 days post-inoculation (dpi), bacteria were recovered from the inoculated leaves. Bacterial recovery was carried out by taking five 10-mm-diameter discs with a cork-borer, which were homogenized by mechanical disruption into 1 ml of 10 mM MgCl_2_. Bacterial enumeration was performed by serial dilution and plating of the samples onto agar plates with cycloheximide and the appropriate antibiotic to differentiate the strains within the mixed infection. For standard replication assays, the same inoculation procedure was carried out using an individual instead of a mixed inoculum.

The CI is defined as the mutant-to-wild type ratio within the output sample divided by the mutant-to-wild type ratio within the input (inoculum) (Freter et al., 1981; Taylor et al., 1987). Mean values are the result of at least three independent experiments with three replicates per experiment. Errors bars represent standard error. Each CI was analyzed using a homoscedastic and 2-tailed Student’s *t*-test and the null hypothesis: mean index is not significantly different from 1 (*P*-value < 0.05).

For macroscopic hypersensitive response (HR) assays and symptom development assays, fully expanded leaves of 10–12-day-old bean plants or 4–5-week-old *Arabidopsis* plants were inoculated using a blunt syringe with a 5 × 10^7^ cfu/ml (for HR) or 5 × 10^5^ cfu/ml (for symptom development) bacterial suspension. HR-derived necrosis was documented at 20 h post-inoculation (hpi) or 24 hpi and disease symptoms were documented at 7 dpi. For standard replication assays, plant leaves were inoculated following the same procedure, and bacteria recovered and analyzed at the indicated times post-inoculation as indicated for CI assays.

For *trans*-complementation assays, one of the strains was inoculated first and, after leaving 2 h for the tissue to recover, the second strain was inoculated covering a partially overlapping area. Both strains were also inoculated individually. All inoculations were carried out at 5 × 10^7^ cfu/ml for visible necrosis to be developed. Leaves were then monitored over time, with photographs taken at different time points to show first the onset of HR and later on the progression of disease symptoms beyond the area inoculated with the overlapping strains. Bacterial recovery to look for bacterial spread beyond the inoculated areas were carried out taking the samples as indicated above in areas neighboring inoculated tissue. Colonies corresponding to each strain were identified by their distinctive morphology on LB plates, and identification was confirmed by replica plating on selective media supplemented with Km.

### Conductivity Assays

To measure cell death induced by *P. syringae* strains during an incompatible interaction associated to the onset of HR in bean, leaves were syringe-infiltrated with a 5 × 10^7^ cfu/ml suspension of the indicated strain and four discs taken per leaf at the indicated time points, and immersed in 6 ml of distilled water for 30 min. To measure cell death induced by *Agrobacterium*-mediated HopZ3 expression in bean, leaves were syringe-infiltrated as indicated below. Ten hours after infiltration, four leaf discs were immersed in 6 ml of distilled water for 30 min. In all assays, leaf discs were then transferred to 6 ml of distilled water and conductivity was measured at the indicated time point using a portable conductivity meter Crison CM35 (Hach-Lange, Barcelona, Spain)

### *Agrobacterium*-Mediated HopZ3-Triggered Immunity Transient Assays

Transient expression assays in bean plants were carried out following the indications described by Vinatzer et al. (2006). Briefly, an overnight culture of *A. tumefaciencs* C58C1 (Supplementary Table S1) carrying the corresponding plasmid (Supplementary Table S2) was diluted into induction medium in a 1:3 proportion and incubated for 5 h at 28°C with shaking. Bacterial cultures were then centrifuged at 10,000 g for 5 min and the pellets re-suspended into infiltration medium. The OD_600_ was adjusted to 0.5 and the strains carrying different vectors were mixed in a 1:1 proportion.

## Results

### HopZ1a Triggers Immunity in Bean

**FIGURE 1:**
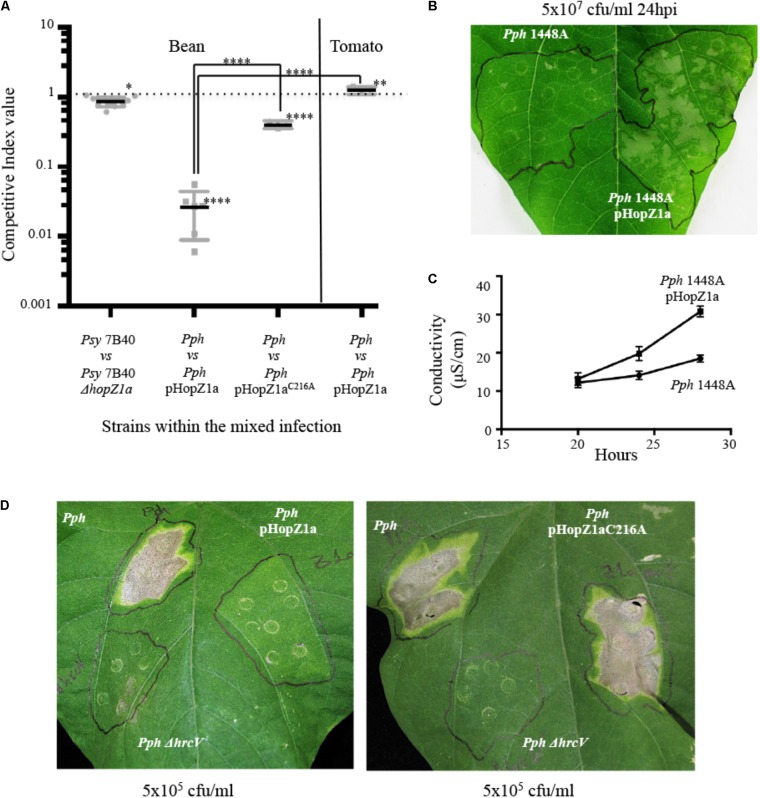
HopZ1a triggers immunity in bean. **(A)** Competitive indices (CIs) measuring proliferation in leaves of *Psy* 7B40 or *Pph* 1448A, either carrying a *ΔhopZ1a* mutation, pHopZa1 (pAME30) or pHopZ1a^C216A^ (pAME27) related to proliferation of *Psy* 7B40 or *Pph* 1448A, as corresponds, in bean or, as indicated. CIs are calculated as the output ratio between the strain lacking or expressing the effector and the corresponding wild type strain, divided by their input ratio. Each CI mean represents at least three independent experiments with three biological replicates each. Individual values are shown for each CI. Error bars represent the standard error. Mean values marked with asterisk(s) were found significantly different from 1.00 or from each other as established by Student’s *t*-test (^∗^*P* < 0.05; ^∗∗^*P* < 0.01; ^∗∗∗∗^*P* < 0.001). **(B)** Hypersensitive response to hand-infiltration of bean leaves with bacterial suspensions containing 5 × 10^7^ cfu/ml of *Pph* 1448A-carrying or not pHopZ1a (pAME30). Photographs were taken 24 h post-inoculation. Image is representative of at least three inoculated leaves per strain and experiment. The experiment was repeated at least twice with similar results. **(C)** Conductivity assays on bean leaves carried out at different time points post-inoculation with 5 × 10^7^ cfu/ml of *Pph* 1448A-carrying or not pHopZ1a (pAME30). The graph shows results from two independent experiments with three inoculated leaves per strain and experiment. **(D)** Disease symptoms to hand-infiltration of bean leaves with bacterial suspensions containing 5 × 10^5^ cfu/ml of *Pph* 1448A-carrying pHopZ1a (pAME30) or pHopZ1a^C216A^ (pAME27), or a *ΔhrcV* mutation. Photographs were taken 7 days post-inoculation. Images are representative of at least three inoculated leaves per strain and experiment. The experiment was repeated at least twice with similar results.

We previously showed that HopZ1a expressed from *P. syringae* pv. *tomato* DC3000 acts as a general suppressor of ETI in *Arabidopsis* (Macho et al., 2010; Rufián et al., 2015). In order to investigate HopZ1a contribution to virulence in its native background, we deleted the *hopZ1a* gene in *P. syringae* pv. *syringae* (hereafter *Psy*) strain 7B40 (Supplementary Table S1) and tested the ability of the mutant to colonize bean leaves using the competitive index (CI) assay (Macho et al., 2007, 2016). We found a small albeit statistically significant attenuation (CI = 0.85 ± 0.035) for the Δ*hopZ1a* mutant strain compared with that of the *Psy* 7B40 wild type (**Figure 1A**). However, population levels for both wild type and mutant strains were considerably smaller than those typically reached by pathogenic *P. syringae* strains within susceptible hosts (Supplementary Figure S1), more in keeping with those reached within resistant hosts. Indeed, a visible necrosis plausibly corresponding to the onset of HR could be detected 24 h after inoculating bean leaves with at 5 × 10^7^ cfu/ml with either *Psy* 7B40 or its Δ*hopZ1a* mutant derivative (Supplementary Figure S1). These results were supported by conductivity assays (Supplementary Figure S1).

Since our group and others had previously shown that HopZ1a suppresses both PTI and ETI, and suppression of ETI is general, with HopZ1a suppressing ETI triggered through independent signaling pathways by different effectors (Macho et al., 2010), we reasoned that HopZ1a could be expected to also suppress bean defenses triggered against other *P. syringae* strains, and thus improve their ability to colonize bean leaves, perhaps providing a clearer phenotype than that obtained from the analysis of the *Psy* 7B40 Δ*hopZ1a* mutant. To assay this, we expressed HopZ1a from pAMEX, a plasmid previously used for the molecular characterization of its virulence function in *Arabidopsis* (Macho et al., 2007, 2010; Rufián et al., 2015) (pAME30, Supplementary Table S2). To our surprise, when this plasmid was used to express HopZ1a in the bean pathogen *P. syringae* pv. *phaseolicola* (hereafter *Pph*) strain 1448A, instead of improving bacterial ability to multiply in bean leaves, it strongly reduced it (20–100-fold decrease) (**Figure 1A**). This effect was mostly eliminated when the plasmid expressed a catalytic mutant version of HopZ1a, HopZ1a^C216A^ (**Figure 1A**), which carries an amino acid change that has been previously shown to impair its virulence function and to prevent activation of HopZ1a-triggered immunity in *Arabidopsis* (Zhou et al., 2009). Furthermore, 24 h after inoculation of *Pph* 1448A carrying the plasmid with 5 × 10^7^ cfu/ml, bean leaves displayed visible necrosis and an increase in conductivity that could correspond with the onset of HR (**Figure 1C**). Additionally, expression of HopZ1a from the plasmid protected bean against infection by *Pph* 1448A, since leaves inoculated with 5 × 10^5^ cfu/ml of *Pph* 1448A carrying pHopZ1a displayed no disease symptoms 7 dpi, looking like those inoculated with a *ΔhrcV* T3SS-defective mutant, while those inoculated with *Pph* 1448A or *Pph* 1448A expressing HopZ1a^C216A^ were fully symptomatic (**Figure 1D**). Plasmid pHopZ1a was stably maintained in *Pph* 1448A, and expression of HopZ1a from this plasmid did not reduce the ability to multiply this strain either in laboratory medium (Supplementary Figure S1) or in tomato (**Figure 1A**), which has been previously shown to lack a resistance gene against this effector (Macho et al., 2010), indicating that the presence of the plasmid does not impact bacterial growth *per se*. Indeed, the presence of the pHopZ1a plasmid caused a slight albeit significant (*P* < 0.01) increase of bacterial proliferation in tomato, supporting the contribution of HopZ1a to virulence in the absence of a defense response. In summary, results obtained for HR, conductivity, and bacterial growth assays in bean leaves inoculated with *Pph* 1448A expressing HopZ1a, in comparison to those obtained for *Pph* 1448A expressing HopZ1a^C216A^, or to those obtained for *Pph* 1448A expressing HopZ1a in tomato, support the notion of HopZ1a activity triggering ETI in bean.

### HopZ1a-Triggered Immunity in Bean Is Not Detected When the Effector Is Expressed From *Psy* 7B40

**FIGURE 2:**
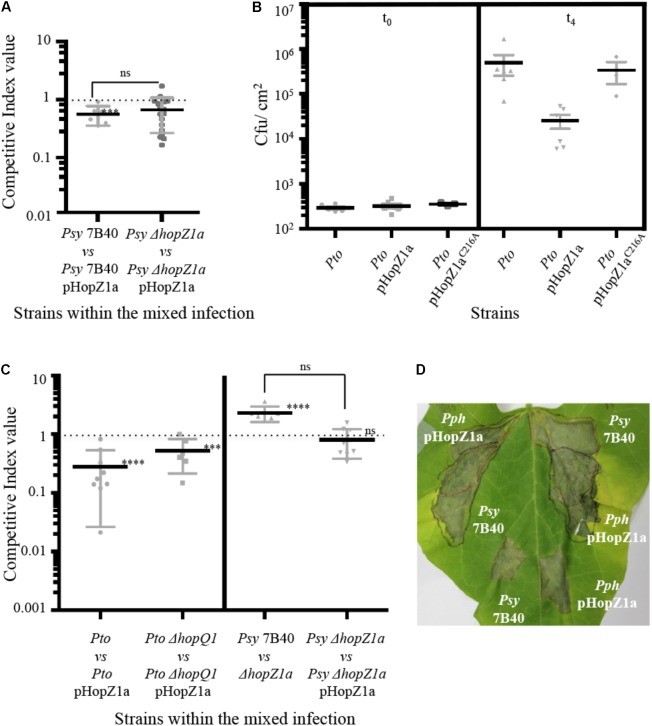
HopZ1a-triggered immunity in bean is suppressed when delivered by *Psy* 7B40. **(A)** Competitive indices (CIs) measuring bacterial proliferation in bean of *Psy* 7B40 or *Psy* 7B40 *ΔhopZ1a-*carrying pHopZ1a (pAME30), related to proliferation of *Psy* 7B40 or *Psy* 7B40 *ΔhopZ1a*, respectively. CIs are calculated as the output ratio between the strain expressing the effector and the corresponding wild type or mutant strain, divided by their input ratio. Leaves were infiltrated with a (1:1) 5 × 10^4^ cfu/ml bacterial suspension. Each CI mean represents the means of at least three independent experiments with three biological replicates each. Individual values are shown for each CI. Error bars represent the standard error. Mean values marked with asterisk(s) were found significantly different from 1.00 or from each other as established by Student’s *t*-test (^∗∗∗^*P* < 0.005; ns not significant). **(B)** Bacterial proliferation within bean leaves. Bean leaves were inoculated by infiltration with bacterial suspensions containing 5 × 10^4^ cfu/ml of *Pto* DC3000, *Pto* DC3000-carrying pHopZa1 (pAME30) or *Pto* DC3000-carrying pHopZ1a^C216A^ (pAME27). Bacterial loads were determined immediately after inoculation or 4 days post-inoculation (dpi). Individual values are shown. Error bars represent standard error. Smallest error bars may be covered by the mean and or individual symbols. **(C)** CIs measuring bacterial proliferation in *N. benthamiana* of *Pto* DC3000, *Pto* DC3000 *ΔhopQ1*, or *Psy* 7B40 *ΔhopZ1a*-carrying pHopZ1a (pAME30), related to proliferation of the corresponding strain without the plasmid, and *Psy* 7B40 *ΔhopZ1a* in relation to that of *Psy* 7B40. CIs are calculated as the output ratio between the strain expressing the effector and the corresponding wild type or mutant strain, divided by their input ratio. Leaves were infiltrated with a 5 × 10^5^ cfu/ml bacterial suspension (2.5 × 10^5^ cfu/ml of each strain, a 1:1 ratio). Each CI mean represents the means of at least three independent experiments with three biological replicates each. Individual values are shown for each CI. Error bars represent the standard error. Mean values marked with asterisk(s) were found significantly different from 1.00 or from each other as established by Student’s *t*-test (^∗∗∗^*P* < 0.005; ^∗∗∗∗^*P* < 0.001; ns not significant). **(D)** Bean leaves infiltrated with 10^7^ cfu/ml of *Psy* 7B40 or *Pph* 1448A-carrying pHopZ1a (pAME30) either separately (bottom) or covering overlapping areas (top). In the later case, one infiltration was carried out first followed by the second infiltration 2 h later. *Pph* 1448A-carrying pHopZ1a (pAME30) was infiltrated first (top left) or second (top right) to rule out differences due to inoculation order. Photographs were taken 8 dpi. Each experiment included at least three replicates. Experiments were repeated at least twice with similar results.

Results shown above support the conclusion that HopZ1a triggers immunity in bean when delivered from *Pph* 1448A. Thus, we would have expected that deleting *hopZ1a* from its native *Psy* 7B40 strain should have improved its ability to multiply in bean, instead of reducing it. To gather additional information, we analyzed the impact of expressing HopZ1a from pHopZ1a in both *Psy* 7B40 and the *Psy* 7B40 Δ*hopZ1a* mutant. *Psy* 7B40-carrying pHopZ1a displayed a slightly reduced ability to multiply within bean leaves compared with the strain without the plasmid (CI statistically different from 1.0), but such an effect was not significant for *Psy* 7B40 Δ*hopZ1a*-carrying pHopZ1a (CI not significantly different from 1.0), nor was the difference between the CIs obtained for these two strains significant (**Figure 2A**). Moreover, the attenuation obtained for these two strains carrying the pHopZ1a plasmid was not significantly different from that caused by the Δ*hopZ1a* mutation. Hence, differences between expressing HopZ1a from its native promoter, or a constitutive promoter, or not expressing it at all were indeed difficult to significantly establish in *Psy* 7B40, in clear contrast with results obtained in *Pph* 1448A (**Figure 1**). However, because the ability of Psy 7B40 to multiply in bean was restricted by HopZ1a-independent immunity (Supplementary Figure S1), a potential caveat was that an additional reduction associated to constitutive expression of HopZ1a from pHopZ1a could perhaps be difficult to detect. To rule out this possibility, we analyzed the impact of expressing HopZ1a on the ability of a non-host strain for bean, *P. syringae* pv. *tomato* DC3000 (hereafter *Pto*), to multiply. Expression of HopZ1a from *Pto* DC3000 carrying this plasmid had been previously shown to induce ETI in *Arabidopsis* (Macho et al., 2010). Bacterial populations of *Pto* DC3000 carrying the pHoZ1a plasmid in bean leaves 4 dpi with 5 × 10^5^ cfu/ml were on average 20-fold smaller that those reached by *Pto* DC3000 not carrying the plasmid (**Figure 2B**), and this reduction was dependent on the integrity of the catalytic site of HopZ1a. This attenuation is in clear contrast with the results obtained for these strains in tomato (Rufián et al., 2018), which lacks a resistance gene against HopZ1a (Macho et al., 2010) and where all three strains multiply to similar levels.

Since *A. tumefaciens-*mediated transient expression of HopZ1a from a 35S promoter induces the HR in the model plant *N. benthamiana* (Ma et al., 2006; Zhou et al., 2009; Rufián et al., 2015), we also carried out CI assays within *N. benthamiana* leaves using *Pto* DC3000-carrying vs. not carrying pHopZ1a and *Pto* DC3000 Δ*hopQ1-*carrying vs. not carrying the plasmid (**Figure 2C**). The effector HopQ1 triggers HR in *N. benthamiana* and a *Pto* DC3000 Δ*hopQ1* mutant has been described to be fully pathogenic in this plant species (Wei et al., 2007). The CIs obtained in both cases indicated that expression of HopZ1a from *Pto* DC3000 causes a significant attenuation in *N. benthamiana* (significantly different from 1; **Figure 2C**), in keeping with HopZ1a triggering HR in a resistant plant when delivered from *Pto* DC3000.

We also analyzed the impact of HopZ1a on the ability of *Psy* 7B40 to multiply in *N. benthamiana.* In this host, the Δ*hopZ1a* mutation determined an improvement in bacterial replication (CI = 2.29 ± 0.277, significantly different from 1; **Figure 2C**). This result further confirms that the hopZ1a gene is expressed and the effector effectively translocated in *Psy* 7B40. Constitutive expression of HopZ1a from the plasmid reduced multiplication of the mutant strain to wild type levels (CI not significantly different from 1), although the two CIs were not significantly different from each other (**Figure 2C**). As observed in bean, a clear ETI is triggered in *N. benthamiana* against HopZ1a when delivered by *Pto* DC3000, however, in this host HopZ1a seems to have only a slight negative effect in virulence when delivered from *Psy* 7B40. This suggests that HopZ1a triggers a weaker defense response in *N. benthamiana* when delivered from this strain than when delivered by the heterologous strain *Pto* DC3000.

Results so far show that HopZ1a triggers strong immunity when delivered from strains that do not natively encode this effector, but triggers a weaker immunity or no immunity at all when delivered from its native background. One plausible explanation for these results is that in bean, and to a lesser extent in *N. benthamiana*, R proteins trigger immunity upon detection of the activity of HopZ1a, and *Psy* 7B40-encoded proteins, perhaps T3Es, are capable of fully or partially suppressing this immunity.

In order to investigate this hypothesis, we analyzed the ability of *Psy* 7B40 to suppress *in trans* the virulence attenuation caused by the expression of HopZ1a in *Pph* 1448A, in bean plants. Control inoculations of bean leaves using 10^7^ cfu/ml of either *Pph* 1448A*-*carrying pHopZ1a, or *Psy* 7B40 without the plasmid, lead to the rapid development of necrosis (**Figure 2D**; bottom of the leaf; Supplementary Figure S2), with no chlorosis or disease symptoms whatsoever spreading from the inoculated area. When both strains are inoculated covering overlapping areas of the leaf (**Figure 2D**; top of the leaf; Supplementary Figure S2) rapid development of necrosis also takes place (24 hpi with *Psy* 7B40 and 24–36 hpi for *Pph* 1448A-carrying pHopZ1a), however at later time points (8 dpi onwards) chlorosis and disease symptoms spread from the overlapping area to non-infiltrated neighboring parts of the leaf (**Figure 2D**). Indeed, if left longer, the disease spreads until the whole leaf first becomes chlorotic, then necrotic, and finally falls (Supplementary Figure S2), suggesting that resistance has been overcome where the two strains are simultaneously present. Only *Pph* 1448A was isolated from leaf samples taken from neighboring non-inoculated areas before disease symptoms appeared (Supplementary Figure S2), demonstrating that symptom progression beyond areas inoculated with the two strains is preceded by *Pph* 1448A spreading, as expected from *bona fide* disease progression. No plasmid loss is detected amongst the population of *Pph* 1448A recovered from these areas, since replica plating shows that 100% of the *Pph* 1448A clones isolated in LB plates display resistance to Km. *Psy* 7B40 was not detected outside the co-inoculated areas. Thus, *Pph* 1448A overcomes HopZ1a-triggered ETI and is capable of causing disease in bean when co-inoculated with *Psy* 7B40. These results support the notion of *Psy* 7B40 encoding an activity, likely an effector(s), capable of suppressing HopZ1a-meditated immunity.

### Heterologous Delivery of HopZ1b Triggers Immunity

Interestingly, similar analysis carried out by our laboratory with another allelic variant of HopZ1a, HopZ1c (Ma et al., 2006), originally identified in the tomato pathogen *P. syringae* pv. *maculicola* ES4326 (hereafter *Pma* ES4326), provided similar results (Rufián et al., 2018). A small although significant attenuation of bacterial proliferation was reported for the *P. syringae* pv. *maculicola* ES4326 *ΔhopZ1c* mutant strain in tomato, which was complemented by expression of HopZ1c from a plasmid. However, expression of HopZ1c from this plasmid caused a significant attenuation of bacterial colonization of tomato leaves in *Pto* DC3000, which does not encode any HopZ effector, suggesting that, as in the case of HopZ1a, HopZ1c-triggered defenses are suppressed by additional effector(s) from within the same effector repertoire in their original pathosystems. Thus, we decided to test whether this could also be the case for the remaining allelic variant, HopZ1b. HopZ1b is encoded in several *P. syringae* pv. *glycinea* strains, including UnB647 (hereafter *Pgy* UnB647). Although *Pgy* UnB647 clusters with soybean pathogenic strains (Ma et al., 2006), this strain was originally isolated from kidney bean (like *Pph* 1448A) and is pathogenic in this host (Sarkar and Guttman, 2004; Hwang et al., 2005; Ma et al., 2006).

**FIGURE 3:**
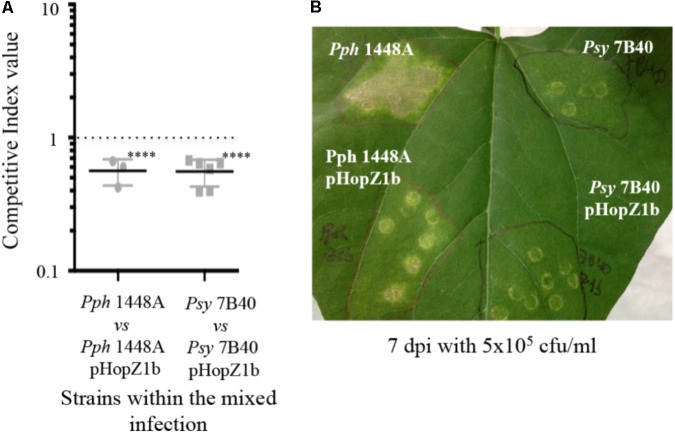
HopZ1b triggers immunity in bean when delivered by either *Pph* 1448A or *Psy* 7B40. **(A)** Competitive indices (CIs) measuring bacterial proliferation in bean of *Pph* 1448A or *Psy* 7B40-carrying pHopZ1b (pJRU6, Supplementary Table S2), in relation to proliferation of the corresponding strain without the plasmid. CIs are calculated as the output ratio between the strain expressing the effector and the corresponding wild type or mutant strain, divided by their input ratio. Each CI mean represents at least the means of three independent experiments with three biological replicates each. Individual values are shown for each CI. Error bars represent the standard error. Mean values marked with asterisk(s) were found significantly different from 1.00 or from each other as established by Student’s *t*-test (^∗∗∗∗^*P* < 0.001; ns not significant). **(B)** Disease symptoms to hand-infiltration of bean leaves with bacterial suspensions containing 5 × 10^5^ cfu/ml of *Pph* 1448A or *Psy* 7B40-carrying or not carrying pHopZ1b (pJRU6). Photographs were taken 7 days post-inoculation. Images are representative of at least three inoculated leaves per strain and experiment. The experiment was repeated at least twice with similar results.

Although we could not carry out a full examination of the effects of either mutation or constitutive expression of HopZ1b in *Pgy* UnB647, since we failed to transform this strain, we did find that expression of HopZ1b from a plasmid determines a reduction of bacterial populations of *Pph* 1448A (CI = 0.562 ± 0.062; **Figure 3A**) and a reduction in the induction of disease symptoms (**Figure 3B**). Expression of HopZ1b from a plasmid also caused a reduction of bacterial colonization of bean leaves in *Psy* 7B40 (CI = 0.479 ± 0.082) (**Figure 3A**), showing that even though this strain multiplies poorly in kidney bean, it can still be further attenuated by the ETI triggered by a plasmid-encoded effector, HopZ1b in this case, further supporting the notion presented above that immunity against plasmid-encoded HopZ1a is suppressed in this strain. Since HopZ1a and HopZ1b alleles have been reported to trigger different resistance pathways (Zhou et al., 2009), the ability of *Psy* 7B40 to suppress HopZ1a-mediated immunity was not expected to result in suppression of HopZ1b-mediated responses.

### Searching for a Suppressor of HopZ1a-Triggered Immunity in *Psy* 7B40

We have previously used plasmid-mediated bacterial co-expression of HopZ1a and other ETI-triggering effector genes, in the form of a bicistronic transcriptional unit generated from a single promoter, to demonstrate HopZ1a ETI suppression abilities (Macho et al., 2010; Rufián et al., 2015). Using these technical setting as a basis, we generated a library of 3–5 kb DNA fragments resulting from a partial Sau3AI digestion, covering the *Psy* 7B40 genome, cloned downstream *hopZ1a* in pAME30 (Supplementary Table S2), as a polycistron under the control of the P*nptII* promoter. The resulting plasmid library was transformed into *Pph* 1448A eYFP (Supplementary Figure S3). Pools of 1,000-transformant clones were co-inoculated into bean leaves at 5 × 10^5^ cfu/ml and YFP fluorescence used to follow bacterial replication within the plant. Control leaves inoculated with 5 × 10^5^ cfu/ml of *Pph* 1448A eYFP pHopZ1a (pAM30, Supplementary Table S2, empty vector for the purposes of the candidate suppressor library) displayed small yellow spots under the fluorescence microscope 5 dpi, clearly different from the larger yellow areas displayed by leaves inoculated with 5 × 10^5^ cfu/ml 1448A eYFP at the same time point post-inoculation (Supplementary Figure S4). Therefore, the extension of the fluorescent areas was in direct correlation with bacterial multiplication in the leaves, with bacteria triggering ETI as a result of HopZ1a expression being confined to small spots. An additional control was carried out using 1:1,000 mix of 1448A eYFP pHopZ1a^C216A^:1448A eYFP pHopZ1a. Under the fluorescence stereomicroscope, larger yellow areas were found amongst smaller spots (Supplementary Figure S4). These were carefully dissected with a scalpel and used to recover bacteria that were confirmed to carry pHopZ1a^C216A^. This strategy was followed to screen the library to saturation, that is, larger microcolonies observed in leaves inoculated with any given 1,000-transformant pool were carefully dissected and used to recover bacteria. These recovered bacteria were used to re-inoculate plants repeating the procedure. However, clones recovered from the screen for their ability to develop into large areas displaying eYFP fluorescence, thus potential candidates to carry HopZ1a-suppressing genes were found to carry a re-organized version that has totally or partially lost the *hopZ1a* gene. Considering that the plasmid is stable during growth in the laboratory medium (Supplementary Figure S1), and undetected technical problems or designs flaws notwithstanding, these negative results would suggest that suppression of the strong ETI triggered by HopZ1a might require the action of two or more genes, working in concert or adding quantitatively to the suppression phenotype, and that under the conditions of our screening no single clone displayed detectable suppression, with only rare re-reorganization events being selected.

### HopZ3-Triggered Immunity in Bean Is Suppressed When Expressed From *Psy* B728a

Like HopZ1a, HopZ3 is another member of the HopZ family, originally identified in *Psy* B728a, for which the ability to suppress ETI has been reported (Vinatzer et al., 2006; Lee et al., 2015). Interestingly, HopZ3 is one of the very few T3Es for which suppression abilities have been demonstrated on the ETI triggered by effectors from the same strain, that is, on the immunity triggered in *N benthamiana* by *Psy* B728a effectors AvrPto1, HopAA1, HopAE1, and HopM1 (Vinatzer et al., 2006) and in *Arabidopsis* by AvrB3 and AvrRpm1 (Lee et al., 2015). Although there is evidence indicating that this ETI suppression activity takes place in bean, *Psy* B728a natural host, there is also evidence of a quantitative avirulence activity of HopZ3 in this host plant, since it elicits cell death when transiently expressed in bean using *A. tumefaciens-*mediated assays (Vinatzer et al., 2006).

**FIGURE 4:**
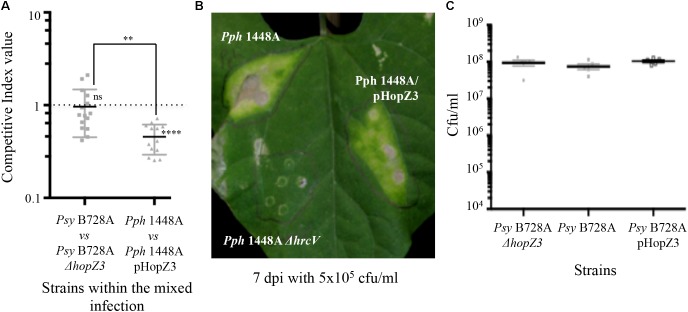
HopZ3 triggers immunity in bean when delivered from *Pph* 1448A but not when delivered from its native strain *Psy* B728a. **(A)** Competitive indices (CIs) measuring bacterial proliferation in bean of *Psy* B728a *ΔhopZ3* or *Pph* 1448A-carrying pHopZ3 (pCMG20, Supplementary Table S2) in relation to proliferation of *Psy* B728a or *Pph* 1448A, respectively. CIs are calculated as the output ratio between the strain expressing the effector and the corresponding wild type or mutant strain, divided by their input ratio. Each CI mean represents the means of at least three independent experiments with three biological replicates each. Individual values are shown for each CI. Error bars represent the standard error. Mean values marked with asterisk(s) were found significantly different from 1.00 or from each other as established by Student’s *t*-test (^∗∗^*P* < 0.01; ns not significant). **(B)** Disease symptoms to hand-infiltration of bean leaves with bacterial suspensions containing 5 × 10^5^ cfu/ml of *Pph* 1448A, or *Pph* 1448A carrying either pHopZ3 (pCMG20) or a *ΔhrcV* mutation. Photographs were taken 7 days post-inoculation (dpi). Images are representative of at least three inoculated leaves per strain and experiment. The experiment was repeated at least twice with similar results. **(C)** Bacterial proliferation within bean leaves. Bean leaves were inoculated by infiltration with bacterial suspensions containing 5 × 10^4^ cfu/ml of *Psy* B728a Δ*hopZ3, Psy* B728a, or *Psy* B728a-carrying pHopZ3 (pCMG20). Bacterial loads were determined 4 dpi. Individual values are shown. Error bars represent standard error. Smallest error bars may be covered by the mean and or individual symbols.

In keeping with the notion of HopZ3 eliciting immunity in bean, delivery of HopZ3 from *Pph* 1448A significantly reduced bacterial ability to colonize bean leaves (**Figure 4A**). Furthermore, the onset of disease symptoms in bean leaves 7 dpi with 5 × 10^5^ cfu/ml of *Pph* 1448A expressing HopZ3 was consistently delayed compared with that of leaves inoculated with *Pph* 1448A (**Figure 4B**). However, unlike Vinatzer and collaborators (2006) who found that the *Psy* B728a Δ*hopZ3* mutant derivative caused increased disease symptoms and displayed increased colonization in snap bean, we did not detect any significant change in the ability to colonize the intercellular spaces of bean leaves of *Psy* B728a *ΔhopZ3* using either competitive assays or individual bacterial multiplication assays (**Figures 4A,C**). We also found no significant differences between *Psy* B728a-expressing, or not, HopZ3 from a plasmid (**Figure 4C**). However, the fact that we used kidney vs. snap bean (Canadian Wonder vs. Blue Lake cultivars), and quantified bacterial growth at later time points (4 dpi vs. 2 dpi), could explain this difference. Indeed Vinatzer and collaborators (2006) reported different roles for *Psy* B728a effectors in different susceptible hosts, and it has also been shown that different bean cultivars display qualitative differences in resistance/susceptibility for many *P. syringae* strains (Hirano and Upper, 2000). Thus, our results indicate that HopZ3 activates stronger defenses in common bean when delivered from *Pph* 1448A than from *Psy* B728a. These results suggest that, as established above for HopZ1a, HopZ3 triggers immunity that could be suppressed by other determinant(s), perhaps T3E(s) from its effector repertoires. Indeed, Vinatzer and collaborators (2006) previously proposed a similar scenario also taking place in *Psy* B782A, with HopZ3 and HopAB1 as suppressors for cell-death eliciting effectors encoded by this strain.

### Searching for a Suppressor of HopZ3-Triggered Immunity in *Psy* B728a

**FIGURE 5:**
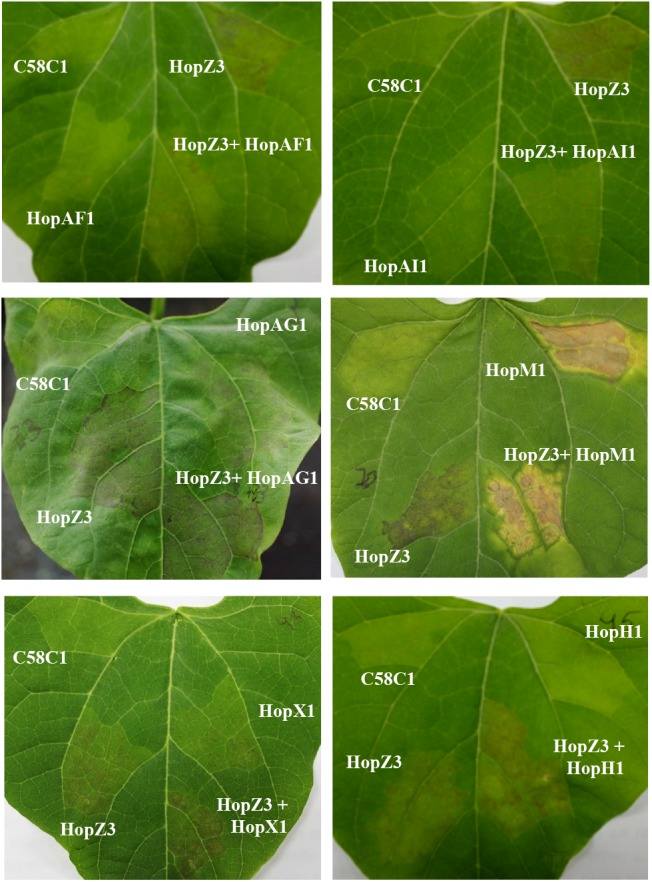
Screening for putative suppressors of HopZ3-triggered immunity in bean. Plant response displayed in bean leaves 48 hours post-inoculation (hpi) with *Agrobacterium tumefaciens* C58C1 carrying binary plasmids containing the genes encoding the indicated effector. Where two effectors are indicated leaves were infiltrated with a 1:1 mixture of *A. tumefaciens* C58C1 carrying a binary plasmid containing the gene encoding each of the indicated effectors. Pictures were taken 48 hpi. The experiment was repeated at least five times with similar results.

As reported for snap bean (Vinatzer et al., 2006), transient *A. tumefaciens*-mediated expression of HopZ3 causes necrosis in kidney bean consistent with the onset of HR (**Figure 5A**), even when we could not detect accumulation of the protein by western blot analysis. Using the strategy previously followed to establish HopZ3-mediated ETI suppression in *N. benthamiana* (Vinatzer et al., 2006), we carried out forward screening looking for effectors from *Psy* B728a with the ability to suppress HopZ3-triggered cell death in kidney bean. As expected, expression of some but not all *Psy* B728a effectors elicited necrosis in kidney bean leaves. Different effectors elicited necrosis to different degrees, which could be occasionally accompanied by chlorosis (**Figure 5** and **Table 1**). Four effectors, AvrRpm1, HopAA1, HopAB1, and HopAE1, had no visible impact on bean responses when either analyzed individually or in combination with HopZ3, however, since we could not establish their expression by western blot analysis these results were classified as non-conclusive (**Table 1**).

**Table 1:** *Psy* B728a type III effectors tested for their ability to suppress HopZ3-dependent necrosis in bean elicited in *Agrobacterium tumefasciens*-mediated transient expression assays.

Type III effectors	Individual symptoms	Suppression
HopAA1	No symptoms	Nc
HopI1	No symptoms	III
HopAB1	No symptoms	Nc
HopJ1	No symptoms	III
HopAG1	Weak to mild necrosis	IV
HopM1	Strong chlorosis and strong necrosis	IV
HopAH1	No symptoms	II
HopAH2	No symptoms	II
AvrB3	Strong necrosis	IV
AvrRpm1	No symptoms	Nc
HopH1	No symptoms	IV
HopX1	Mild necrosis	IV
AvrPto1	No symptoms	II
HopAE1	No symptoms	Nc
HopAI1	No symptoms	II
HopAK1	No symptoms	III
HopAF1	No symptoms	I

*^*a*^Class I, display clear suppression (partial to complete); Class II, display variable suppression (from no suppression to strong suppression); Class III, some replicates show partial suppression; Class IV, no evidence of suppression observed. Nc: non-conclusive results since effectors display no impact on bean defenses when either expressed individually or in combination with HopZ3 and no evidence of accumulation could be established by western blot analyses.*

To differentiate between different levels of cell death suppression activity displayed by different effectors, we classified them into suppressor classes as previously done by Guo and collaborators for *Pto* DC3000 effectors (Guo et al., 2009): Class I effectors included those that displayed clear and reproducible suppression (partial to complete) of HopZ3-triggered necrosis in all replicate experiments, Class II effectors displayed variable ability to suppress (from no suppression to strong suppression) in different replicates, Class III effectors displayed partial suppression in some of the replicates, and Class IV effectors did not display suppression abilities in any of the replicates. One effector was classified into Class I (HopAF1), four were classified into Class II (AvrPto1, HopAH1, HopAH2, and HopAI1), three into Class III (HopAK1, HopI1, and HopJ1), and the remaining five into Class IV (HopAG1, HopM1, AvrB3, HopH1, and HopX1) (**Table 1**).

When expressed in combination with HopZ3, Class IV effectors displayed a variety of outcomes. Remarkably, HopM1 expression caused a very strong necrosis surrounded by a marked chlorosis, regardless of whether it was expressed alone or in combination with HopZ3 (**Figure 5**). The epistasis observed in bean for HopM1-mediated over HopZ3-mediated responses is noteworthy since HopZ3 suppresses HopM1-mediated responses in *N. benthamiana* (Vinatzer et al., 2006). Expression of AvrB3 elicited strong necrosis in kidney bean, very similar to that triggered by HopZ3 (data not shown), although it does not do so when, similarly, expressed in snap bean (Vinatzer et al., 2006), showing these two bean cultivars can display differences in their response to a given effector, as mentioned above. In the case of HopX1 and HopH1, they both lead to stronger necrosis when expressed in combination with HopZ3 than when either of them or HopZ3 were expressed individually, thus displaying a quantitative additive effect on the activation of plant responses in bean (**Figure 5**).

**FIGURE 6:**
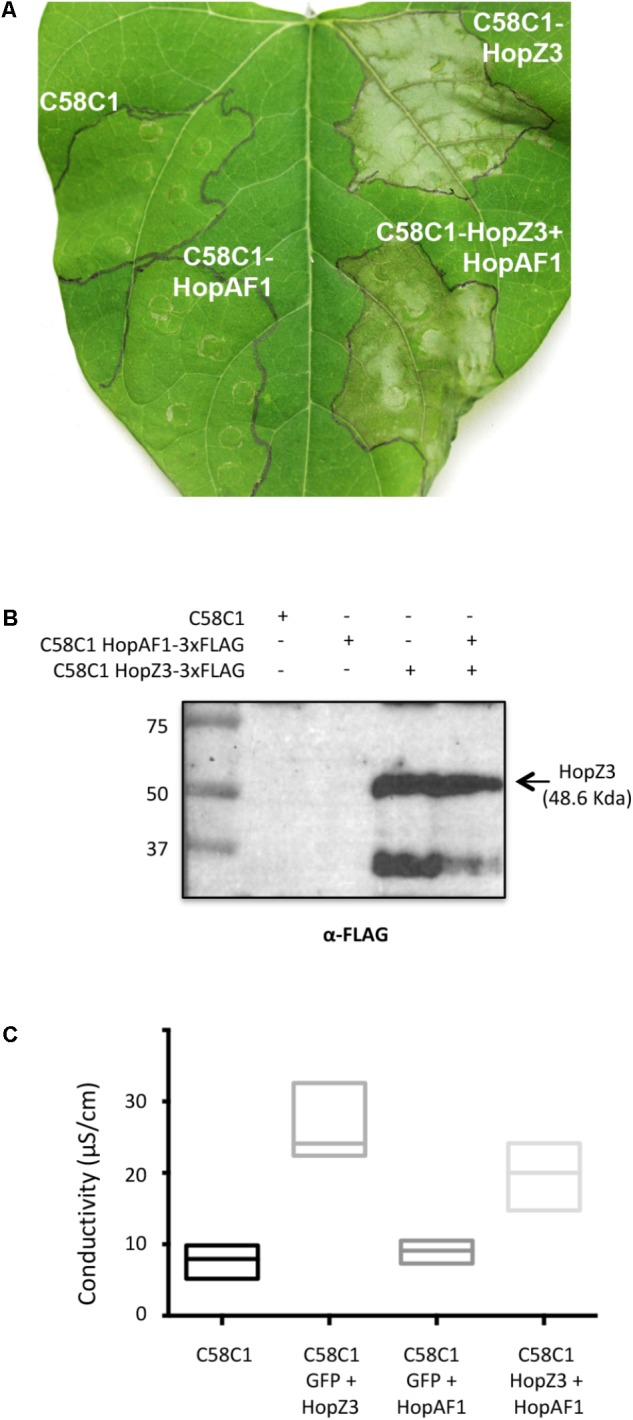
HopAF1 displays suppression activity of HopZ3-triggered immunity in bean. **(A)** Plant response displayed in bean leaves inoculated with *Agrobacterium tumefaciens* C58C1 carrying either or both binary plasmids encoding HopAF1-3xFLAG and HopZ3-3xFLAG. Where two effectors are indicated leaves were infiltrated with a 1:1 mixture of *A. tumefaciens* C58C1 carrying the plasmids encoding each of the indicated effectors. Pictures were taken 48 h post-inoculation. The experiment was repeated three to five times with similar results. **(B)** Western blot analysis of samples taken from bean leaves described in **(A)** using an anti-FLAG antibody. **(C)** Conductivity assays on bean leaves inoculated with *A. tumefaciens* C58C1 or C58C1 carrying binary plasmids encoding GFP-3xFLAG, HopAF1-3xFLAG, or HopZ3-3xFLAG. Where two effectors are indicated leaves were infiltrated with a 1:1 mixture of *A. tumefaciens* C58C1 carrying the plasmids encoding the indicated protein. The graph shows results obtained for three replicate plants per condition 48 hpi.

The fact that accumulation of HopZ3 could not be detected by western blot analysis even when necrosis was observed following HopZ3 individual expression or with co-expression with effectors that did not display suppression, did not allow us to disregard the potential caveat that the suppression observed upon co-expression with some of the effectors could be a consequence of interferences with HopZ3 expression. To rule out this possibility, and as a validation of the results obtained in the screening, we repeated co-expression assays with Class I effector HopAF1 using a 3xFLAG version of HopZ3. Images show suppression of HopZ3-induced necrosis when both effectors were co-expressed, which was supported by additional conductivity assays (**Figure 6**). Western blot analysis failed to detect accumulation of HopAF1 but clearly showed that HopZ3 accumulated to similar levels when expressed alone, inducing necrosis, and when co-expressed with HopAF1, where HR was suppressed, thus confirming HopAF1 as a Class I suppressor of HopZ3-triggered immunity.

Thus, we have found at least eight effectors that display the ability to suppress immunity triggered against HopZ3 in transient expression assays in kidney bean, and are therefore candidates to suppress HopZ3 immunity when delivered from *Psy* B782a, its native strain.

## Discussion

Our results provide evidence of the presence of a resistance gene against HopZ1a in kidney bean, a species of agronomical interest. Since HopZ1a immunity in *Arabidopsis* has been shown to require the function of ZED1 and ZAR1 genes (Lewis et al., 2010, 2013), HopZ1a-triggered immunity in bean plants might be an indication that a ZED1/ZAR1 functional homolog is present in this species. The presence of such a defense complex in bean might not be entirely surprising, considering that although HopZ1a expression does not trigger immunity in tomato (Macho et al., 2010; Rufian et al., 2016), it triggers HR in soybean, *Arabidopsis, N. benthamiana*, rice and sesame (Ma et al., 2006). It was somewhat unexpected though, since bean is a host for a large number of *Psy* strains (Agrios, 2005), and *hopZ1a* has only been found to date in *Psy* strains. But strains carrying *hopZ1a* are believed to multiply in hosts lacking a resistance gene against this effector, and *hopZ1a*-carrying strains have been isolated from a number of hosts different from bean (Ma et al., 2006). Thus, perhaps no *hopZ1a*-carrying strain can colonize bean, and *hopZ1a* could have been lost in bean pathogenic lineages, maybe replaced by mutational derivatives that avoid detection by bean defenses, or even substituted by homologs through horizontal transfer, as proposed by Ma and collaborators (2006). Nevertheless, our finding that *Psy* 7B40 has suppressing activities on HopZ1a-mediated immunity provides an alternative means of adaptation: strains carrying *hopZ1a* could avoid detection by the plant immune system, and therefore the selective pressure to lose *hopZ1a* or to select for mutational derivatives, by acquiring the ability to suppress HopZ1a-triggered immunity. This notion, deeply rooted in the zig-zag model proposed by Jones and Dangl (2006) to explain the co-evolution of plant immunity and pathogen virulence, is supported by the widespread occurrence of ETI-suppressing abilities among T3E effectors (Guo et al., 2009), and by the results presented here providing evidence of suppression of immunity triggered by both HopZ1a and HopZ3. This could perhaps be a common evolutionary strategy of the HopZ family since we have shown elsewhere that HopZ1c triggers immunity in tomato when delivered from the heterologous strain *Pto* DC3000, but not when delivered from the HopZ1c-encoding strain *Pma* ES4326 (Rufián et al., 2018). Also, although we could not carry out a full examination of the effects of either mutation or constitutive expression of HopZ1b in *Pgy* UnB647, since we failed to transform this strain, our findings that expression of HopZ1b from a plasmid determines a reduction of bacterial populations of *Pph* 1448A and *Psy* 7B40, and a reduction in the induction of disease symptoms induced by *Pph* 1448A raises the tempting possibility of HopZ1b-triggered immunity in bean also being suppressed within its native background.

It is noteworthy that although intra-strain suppression of ETI has been assumed to take place for many years, it is rarely taken into account as a viable route for host adaptation in hosts carrying effector-matching R genes. HopZ1a constitutes a good example of a T3E that enjoys a very detailed molecular characterization regarding both, virulence and defense elicitation, but this characterization has been performed out of the context of its accompanying T3Es in the same repertoire and native strain. This might have biased our views as to the role that ETI suppression plays in virulence or in the evolution of virulence of *P. syringae*.

The fact that both HopZ1a and HopZ3 are themselves suppressors of ETI (Macho et al., 2010; Lee et al., 2015; Rufián et al., 2015) that could be “masked” by additional ETI-suppressing T3Es coexisting within the same strain, provides a additional explanation for the long known fact that mutation of individual T3Es usually have little to no impact on virulence, a fact long attributed solely to effector functional redundancy (Kvitko et al., 2009; Zumaquero et al., 2010). Indeed, the impact on virulence of deleting a defense-suppressing effector that triggers immunity that may in turn be suppressed by another effector to allow disease development, would be the resultant of the quantitative contribution of the respective defenses activated and suppressed in each case in each host, as much as of the possible existence of functionally redundant additional effectors within the same inventory. Thus, data presented here support the concept that the T3Es’ repertoire of any given pathogen must function as a whole to determine the final outcome of a particular plant-pathogen interaction, and therefore emphasize the interest of complementing functional characterization of T3Es by assaying its biological relevance in the context of the accompanying effector repertoire, and/or in its native strain. However, this often implies overcoming the technical challenges presented by poorly characterized strains or pathosystems, and does not provide the striking phenotypes or straightforward results frequently obtained in heterologous assays. In this sense, this report complements results presented by Vinatzer and collaborators (Vinatzer et al., 2006) in showing the complexity that cross-suppression of ETI between effectors from the *Psy* B728a effector repertoire can reach different hosts.

An interesting corollary of the results obtained from the analysis of the deletion of *hopZ1a* in *Psy* 7B40 in bean, where the ETI triggered by HopZ1a is suppressed, is that the small albeit significant attenuation detected (and complemented by expression from a plasmid of HopZ1a) supports the ability of HopZ1a to suppress plant immunity in its native background, as previously shown in heterologous pathosystems (Macho et al., 2009, 2010; Lewis et al., 2014).

The fact that our screening of the *Psy* 7B40 genomic library screen did not identify T3Es with HopZ1a-specific ETI-suppressing ability can be attributed to: (i) suppression of ETI requiring the combined action of more than one T3E within the *Psy* 7B40 repertoire; or (ii) the suppression of HopZ1a-induced ETI not relying on T3Es but on other polygenic virulence determinants, such as phytotoxins. Phytotoxins have been shown to complement virulence functions of T3E (Melotto et al., 2006). We can rule out phaseolotoxin or coronatin as suppressors of HopZ1a-triggered immunity since *Pph* 1448A and *Pto* DC3000, which produce these toxins, respectively, do not suppress HopZ1a-triggered ETI. The involvement of syringolin, syringopeptin, or syringomycin could seem more likely since the majority of strains natively carrying *hopZ1a* belong to *P. syringae* multilocus sequence typing (MLST) group II (Ma et al., 2006; Baltrus et al., 2011). Further research would be necessary to establish the molecular mechanism behind suppression of HopZ1a-triggered immunity in *Psy* 7B40.

In *Arabidopsis*, HopZ3 suppresses AvrB3-triggered and AvrRpm1-triggered immunity through interaction with these two effectors and their plant targets, included in the RPM1 defense hub (Lee et al., 2015). It has been suggested that the formation of such T3E complexes might facilitate their interference with host components, in this instance promoting HopZ3 suppression of AvrB3-triggered and AvrRpm1-triggered immunity. The possibility that such a multi-protein complex might include additional T3Es contributing to suppress HopZ3-triggered immunity, such as those identified in our forward screening, is an appealing hypothesis. Interestingly and considering that our screening implied high-level co-expression of HopZ3 paired with different T3Es from the *Psy* B728a repertoire, and the fact that HopZ3 interacts with AvrB3 and AvrRpm1, might have led to interference with HopZ3-triggered necrosis when expressed in combination with AvrB3 or AvrRpm1. Although results with AvrRpm1 were inconclusive, the fact that co-expression of AvrB3 and HopZ3 induced a plant response similar to that induced by each of these effectors individually, does not provide evidence of defense suppression or of interference with ETI. If AvrB3 immunity was fully suppressed by HopZ3 in bean as previously shown for other hosts, the necrosis observed upon their combined expression would correspond to HopZ3-triggered immunity. Thus, on this basis we tentatively classified AvrB3 as a Class IV effector (not displaying suppressing activity). Another two Class IV effectors, HopX1 and HopH1, led to enhanced responses when expressed in combination with HopZ3. These two effectors could have a quantitative avirulence contribution in bean, additive to that of HopZ3, although HopH1 contribution would not be sufficient on its own to lead to the appearance of visible necrosis, as reported in other cases (Parker et al., 1993; Gassmann, 2005; Vinatzer et al., 2006).

Five effectors displayed different levels of interference with the cell death elicited by HopZ3: AvrPto1, HopAF1, HopAI1, HopAH1, and HopAH2. AvrPto1, HopAF1, and HopAI1 or some of their homologs from other *P. syringae* pathovars have been functionally characterized and shown to interfere with plant defenses at different levels. In *Pto* DC3000, AvrPto1 has been shown to target pattern recognition receptor (PRRs) to suppress PTI to enhance bacterial virulence (Shan et al., 2000, 2008; Xiang et al., 2008; Xiang et al., 2011), and to display ETI-suppressing abilities, having also been characterized as a Class II suppressor in a similar analysis previously (Guo et al., 2009). Interestingly, although AvrPto1 induced no symptoms in kidney bean or as previously shown in snap bean, it triggers immunity in *N. benthamiana* that is suppressed by HopZ3 (Vinatzer et al., 2006), perhaps by interacting with each other and/or with a defense hub in a similar manner to that shown for HopZ3 in *Arabidopsis* (Lee et al., 2015).

HopAF1 is a widely distributed effector in *P. syringae* (Baltrus et al., 2011), which in *Pto* DC3000 has been shown to function as a deamidase involved in PTI suppression (Washington et al., 2016). HopAF1 is targeted to the plasma membrane through a myristoylation domain and deamidates MTN1 and MTN2 thus inhibiting ethylene biosynthesis induced during PTI in *Arabidopsis* (Washington et al., 2016). Additionally, it has also been reported to suppress the ETI triggered in tobacco by HopAD1 (Castaneda-Ojeda et al., 2017). Similar to the case of AvrPto1, the *Pto* DC3000 HopAF1 effector has also been classified as a Class II suppressor of ETI by Guo and collaborators (Guo et al., 2009). In the same study, the *Pto* DC3000 HopAI1 effector was not found to display suppressing activity on HopA1-triggered HR (Guo et al., 2009). However, HopAI1 has been reported to function as a phosphothreonine lyase that dephosphorylates MPK3, MPK4, and MPK6, and inhibits MPK4 activity, thus inhibiting PTI (Zhang et al., 2007, 2012). MPK4 interacts with and phosphorylates RIN4 (Cui et al., 2010). Interestingly, HopZ3 also interacts with MPK4, although does not seem to acetylate it, and with RIN4, a key component of the RPM1 defense hub (Lee et al., 2015). It must be noted though that in *Psy* B728a, *hopAI1* carries an early STOP codon that would render a smaller truncated version than its homolog in *Pto* DC3000. How this affects the potential for HopAI1 to interfere with cell death elicitation by HopZ3 remains to be determined.

Little is known about HopAH1 and HopAH2, which displayed partial interference with HopZ3-triggered immunity (**Table 1**). HopAH1 has been shown to translocate and to be expressed as part of the HrpL-operon (Schechter et al., 2006; Vinatzer et al., 2006), but has not been characterized functionally. HopAH2 has been shown to translocate in *Psy* B728a, *Pto* DC3000, and *Pph* 1448A, however, based on its HrpL-independent expression is not considered by some authors a T3E (Greenberg and Vinatzer, 2003; Schechter et al., 2006; Vinatzer et al., 2006; Macho et al., 2009). However, this argument is debatable since AvrB4-2, another effector expressed in an HrpL-independent manner, has been shown to quantitatively contribute to virulence of *Pph* 1448A in bean (Zumaquero et al., 2010).

Additional work will be necessary to establish whether the effectors identified as candidate suppressors of HopZ3-mediated immunity do so directly, or interfere with this response indirectly. However, the fact that none of these effectors trigger any visible plant response themselves, does suggest that interference is not an indirect consequence of defenses triggered by these effectors in bean negatively impacting on *Agrobacterium*, as shown for the XopQ/HopQ effectors in *N. benthamiana* (Adlung and Bonas, 2017). The identification of these candidate effectors provide an explanation to the results hereby showing that HopZ3-triggered immunity in bean is suppressed when delivered from *Psy* B728a.

The T3E HopZ family is diverse and widely distributed, and displays remarkable evolutionary dynamism, with a high rate of gene loss and incorporation of divergent alleles by horizontal transfer (Baltrus et al., 2011) suggesting an evolutionary cost on many hosts, but also an important role in virulence across a broad range of host species. Our results suggest that a third evolutionary strategy could be involved in the evolution of this family: the expansion of the T3E inventory with additional T3Es with ETI-suppressing ability, which would “mask” the HopZ immunity-triggering effector from host recognition, as has been described previously for other effectors (Tsiamis et al., 2000; Vinatzer et al., 2006; Szczesny et al., 2010; Lee et al., 2015). The fact that HopZ1a and HopZ3 are themselves T3Es with ETI-suppressing ability adds complexity to the interplay of intra-inventory suppressions that can take place between the different effectors in different hosts, which should be factored in its evolutionary scheme.

## Author Contributions

JR, JR-B, CG, AZ, AM, CB, and JR-A participants in the conception and design of the work and experiments. The acquisition of data and its primary analysis has been the responsibility of JR, AL, JR-B, CG, AZ, AM, IO-M, and GR-A while all authors contributed to the final interpretation of the data. The paper has been drafted by the combined efforts of JR, CB, and JR-A, with additional contribution to the final version by AL, JR-B, and AM, after critical revision. All authors approved the final version sent to the Editor of Frontiers in Plant Sciences for its review, and agree to be accountable for the accuracy and integrity of their respective contributions to the work presented in this paper.

## Conflict of Interest Statement

The authors declare that the research was conducted in the absence of any commercial or financial relationships that could be construed as a potential conflict of interest.
