# Supplementary material for: Suppression of HopZ Effector-Triggered Plant Immunity in a Natural Pathosystem
Source: Front Plant Sci. 2018 Aug 14;9:977. doi: 10.3389/fpls.2018.00977 (PMC6103241; doi:10.3389/fpls.2018.00977)
Supplement: Supplementary file 1 [file Table_1.DOCX]

**Table 1S.** **Strains used and generated in this work.**

| **Strain** | **Name and relevant genotype** | **Reference** |
| --- | --- | --- |
| 1448A | *P. syringae* pv. *phaseolicola* race 6 | Teverson, 1991 |
| 7B40 | *P. syringae* pv. *syringae* wild-type | Sundin and Bender, 1996 |
| B728A | *P. syringae* pv. *syringae* wild-type | Loper and Lindow (1987) |
| DC3000 | *P. syringae* pv. *tomato* Rif^R^ | Cuppels, 1986 |
| C58C1 | *Agrobacterium tumefaciens* | [Deblaere et al., 1985](#_ENREF_7) |
| JRP9 | 1448A eYFP Gm^R^ | Rufián et al., 2017 |
| IOM1 | 1448A *ΔhrcV*, Km^R^ | Macho et al., 2007 |
| AZJ35 | 7B40 *ΔhopZ1a* Km^R^ | This work |
| AZJ36 | B728A *ΔhopZ3* Km^R^ | This work |
| AZJ37 | DC3000 *ΔhopQ1-1* Km^R^ | This work |
