# Supplementary material for: Suppression of HopZ Effector-Triggered Plant Immunity in a Natural Pathosystem
Source: Front Plant Sci. 2018 Aug 14;9:977. doi: 10.3389/fpls.2018.00977 (PMC6103241; doi:10.3389/fpls.2018.00977)
Supplement: Supplementary file 2 [file Table_2.DOCX]

**Table 2S.** Plasmids used and generated in this work

| **Name** | **Description** | **Antibiotic resistance*** | **Reference** |
| --- | --- | --- | --- |
| pAMEX | Expression vector carrying P*nptII* | Amp, Km | Macho *et al*., 2009 |
| pMD1 | Binary vector carrying *35S* promoter, C-term-3xFLAG  (Gateway destination vector) | Km | Li *et al*., 2013 |
| pAME30 | pAMEX derivative carrying P*nptII::hopZ1a* | Amp, Km | Macho *et al*., 2010 |
| pAME27 | pAMEX derivative carrying P*nptII::hopZ1a^C216A^* | Amp, Km | Macho *et al*., 2010 |
| pGEM-T-KmFRT-EcoRI | pGEM-T derivative containing an FRT-flanked *nptII gene* | Amp, Km | Zumaquero *et al*., 2010 |
| pUCP20tk::HopZ1b-HA | Carries *hopZ1b* with its own promoter | Km | Zhou *et al*., 2009 |
| pBAV139-HopZ3 | Binary vector carrying *35S::hopZ3-HA* | Km | Vinatzer *et al*., 2006 |
| pBAV139-HopAA1 | Binary vector carrying *35S::hopAA1-HA* | Km | Vinatzer *et al*., 2006 |
| pBAV139-HopI1 | Binary vector carrying *35S::hopI1-HA* | Km | Vinatzer *et al*., 2006 |
| pBAV139-HopAB1 | Binary vector carrying *35S::hopAB1-HA* | Km | Vinatzer et al., 2006 |
| pBAV139-HopJ1 | Binary vector carrying *35S::hopJ1-HA* | Km | Vinatzer *et al*., 2006 |
| pBAV139-HopAG1 | Binary vector carrying *35S::hopAG1-HA* | Km | Vinatzer *et al*., 2006 |
| pBAV139-HopM1 | Binary vector carrying *35S::hopM1-HA* | Km | Vinatzer *et al*., 2006 |
| pBAV139-HopAH1 | Binary vector carrying *35S::hopAH1-HA* | Km | Vinatzer *et al*., 2006 |
| pBAV139-HopAH2 | Binary vector carrying *35S::hopAH2-HA* | Km | Vinatzer *et al*., 2006 |
| pBAV139-AvrB3 | Binary vector carrying *35S::avrB3-HA* | Km | Vinatzer *et al*., 2006 |
| pBAV139-AvrRpm1 | Binary vector carrying *35S::AvrRpm1-HA* | Km | Vinatzer *et al*., 2006 |
| pBAV139-HopH1 | Binary vector carrying *35S::hopH1-HA* | Km | Vinatzer *et al*, 2006 |
| pBAV139-HopX1 | Binary vector carrying *35S::hopX1-HA* | Km | Vinatzer *et al.*, 2006 |
| pBAV139-AvrPto1 | Binary vector carrying *35S::AvrPto1-HA* | Km | Vinatzer *et al.*, 2006 |
| pBAV139-HopAE1 | Binary vector carrying *35S::hopAE1-HA* | Km | Vinatzer *et al.*, 2006 |
| pBAV139-HopAI1 | Binary vector carrying *35S::hopAI1-HA* | Km | Vinatzer *et al.*, 2006 |
| pBAV139-HopAK1 | Binary vector carrying *35S::hopAK1-HA* | Km | Vinatzer *et al.*, 2006 |
| pBAV139-HopAF1 | Binary vector carrying *35S::hopAF1-HA* | Km | Vinatzer *et al.*, 2006 |
| pAZJ30 | pGEM-T derivative carrying the *∆hopZ1a*::KmFRT knockout allele | Amp, Km | This work |
| pAZJ31 | pGEM-T derivative carrying the *∆hopZ3*::KmFRT knockout allele | Amp, Km | This work |
| pAZJ32 | pGEM-T derivative carrying the *∆hopQ1*::KmFRT knockout allele | Amp, Km | This work |
| pJRU6 | pAMEX derivative carrying P*nptII::hopZ1b* | Amp, Km | This work |
| pCMG20 | pAMEX derivative carrying P*nptII:: shcZ3 hopZ3* | Amp, Km | This work |
| pEARLEYGATE103 | 35S-Gateway-GFP-His | Km | Earley *et al.*, 2006 |
| pMD1-Z3 | Binary vector carrying *35S::hopZ3*::3xFLAG | Km | This work |
| pMD1-AF1 | Binary vector carrying *35S::hopAF1*::3xFLAG | Km | This work |
| pMD1-GFP | Binary vector carrying *35S::gfp*::3xFLAG | Km | This work |

* Amp: Ampicilin. Km: Kanamycin.
