# Supplementary material for: Suppression of HopZ Effector-Triggered Plant Immunity in a Natural Pathosystem
Source: Front Plant Sci. 2018 Aug 14;9:977. doi: 10.3389/fpls.2018.00977 (PMC6103241; doi:10.3389/fpls.2018.00977)
Supplement: Supplementary file 3 [file Table_3.DOCX]

**Table 3S.** Primers used in this work.

| **Name** | **Sequence** | **Restriction site** |
| --- | --- | --- |
| Z1b-F | AAGGATCCGAATAACCTTACCTTCAGG | *Bam*HI |
| Z1b-R | AATCTAGAAGAAACTAGCCCTGAGCCG | *Xba*I |
| Z3-F | AAGAATTCTCCTGACCTGCAGAGTTCC | *Eco*RI |
| Z3-R | AAGGATCCACGTGAGTCAAGGCTTGGC | *Bam*HI |
| Z3Flag-F | AAGCGGCCGCCATGAATATCTCAGGTCCG | *Not*I |
| Z3Flag-R | AAGGCGCGCCCAGGCTTGGCCCGGACCC | *Asc*I |
| Z1aMut-A1 | GATCGTGACGATCTAGTGC | None |
| Z1aMut-A2 | CCCTATAGTGAGTCGAATTCGCCGACGCATACATTTCCC | *Eco*RI |
| Z1aMut-B1 | ATGCTGTCCGAAGCTTGGG | None |
| Z1aMut-B2 | GAATTCGACTCACTATAGGGTACTTGCCGAAGAGCAGCGC | *Eco*RI |
| Z3Mut-A1 | AACCAGATCGCTCACAGCC | None |
| Z3Mut-A2 | CCCTATAGTGAGTCGAATTCAGTCCCCGCCATGGTTAGC | *Eco*RI |
| Z3Mut-B1 | GCTGATGACATGCTTGCG | None |
| Z3Mut-B2 | GAATTCGACTCACTATAGGGAGTCCCCGCCATGGTTAGC | *Eco*RI |
| Q1Mut-A1 | ATGCGCTGGATATCGCTC | None |
| Q1Mut-A2 | CCCTATAGTGAGTCGAATTCTAGTGCCGACAAATGTGG | *Eco*RI |
| Q1Mut-B1 | TAAGCTGCACCTTGCGAC | None |
| Q1Mut-B2 | GAATTCGACTCACTATAGGGCTCCTGGATAGATGAACC | *Eco*RI |
| GFP-F | GCTCCGCGGCCGCCCCCTTCACCATGGTGAGCAAGGGCGAGGAG | *Not*I |
| GFP-R | GCTGGGTCGGCGCGCCCACCCTTCTTGTACAGCTCGTC | *Asc*I |
