# Supplementary material for: Suppression of HopZ Effector-Triggered Plant Immunity in a Natural Pathosystem
Source: Front Plant Sci. 2018 Aug 14;9:977. doi: 10.3389/fpls.2018.00977 (PMC6103241; doi:10.3389/fpls.2018.00977)
Supplement: Supplementary file 4 [file Image_1.PDF]

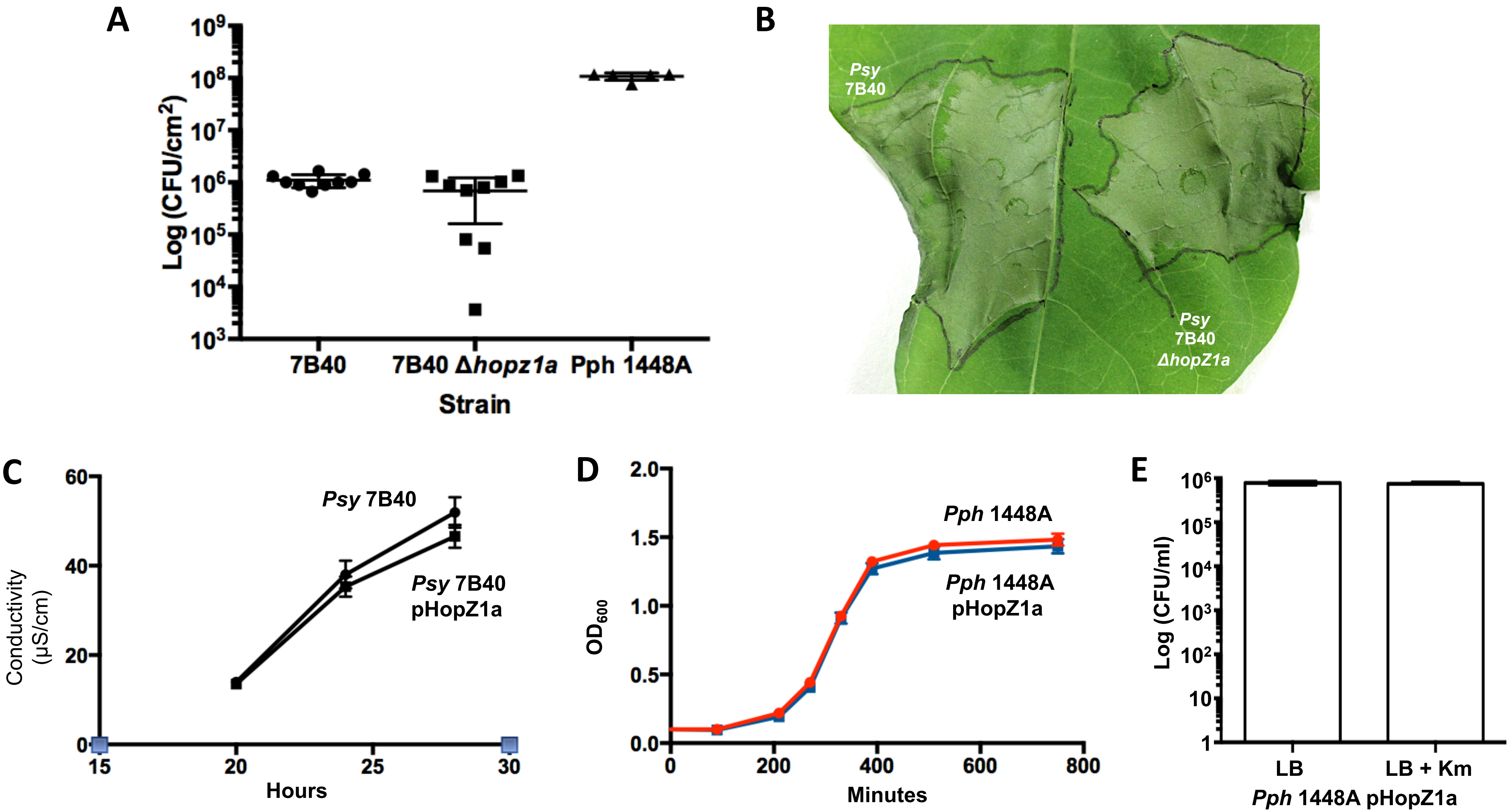


**Figure 1S HopZ1a triggers immunity in bean when delivered from a non-HopZ encoding strain.** (**A**) Proliferation in bean leaves 5 days post-inoculation with $5x10^5$ cfu/ml of *Psy* 7B40, *Psy* 7B40 a *ΔhopZ1a* or *Pph* 1448A corresponding to the individual populations of the competitive indexes shown in Fig. 1A. (**B**) . Imagenes of the hypersensitive response to hand-infiltration of bean leaves with bacterial suspensions containing $5x10^7$ cfu/ml of *Psy* 7B40 carrying or not a *ΔhopZ1a* mutation. Photographs were taken 24 hours post-inoculation. Image is representative of at least 3 inoculated leaves per strain and experiment. The experiment was repeated at least twice with similar results. (**C**) Conductivity assays on bean leaves carried out at different time-points post-inoculation with $5x10^7$ cfu/ml of *Psy* 7B40 carrying or not a *ΔhopZ1a* mutation. The graph shows results from two independent experiments with three inoculated leaves per strain and experiment. (**D**) Bacterial growth curves in LB medium of *Pph* 1448A carrying or not pHopZ1a. The graph shows results obtained from two independent experiments (**E**) Bacteria CFU in LB-grown cultures from D, diluted down to $5x10^3$ and plated onto in LB and LB supplemented with kanamicin plates to test plasmid stability.
