# Supplementary material for: Suppression of HopZ Effector-Triggered Plant Immunity in a Natural Pathosystem
Source: Front Plant Sci. 2018 Aug 14;9:977. doi: 10.3389/fpls.2018.00977 (PMC6103241; doi:10.3389/fpls.2018.00977)
Supplement: Supplementary file 5 [file Image_2.PDF]

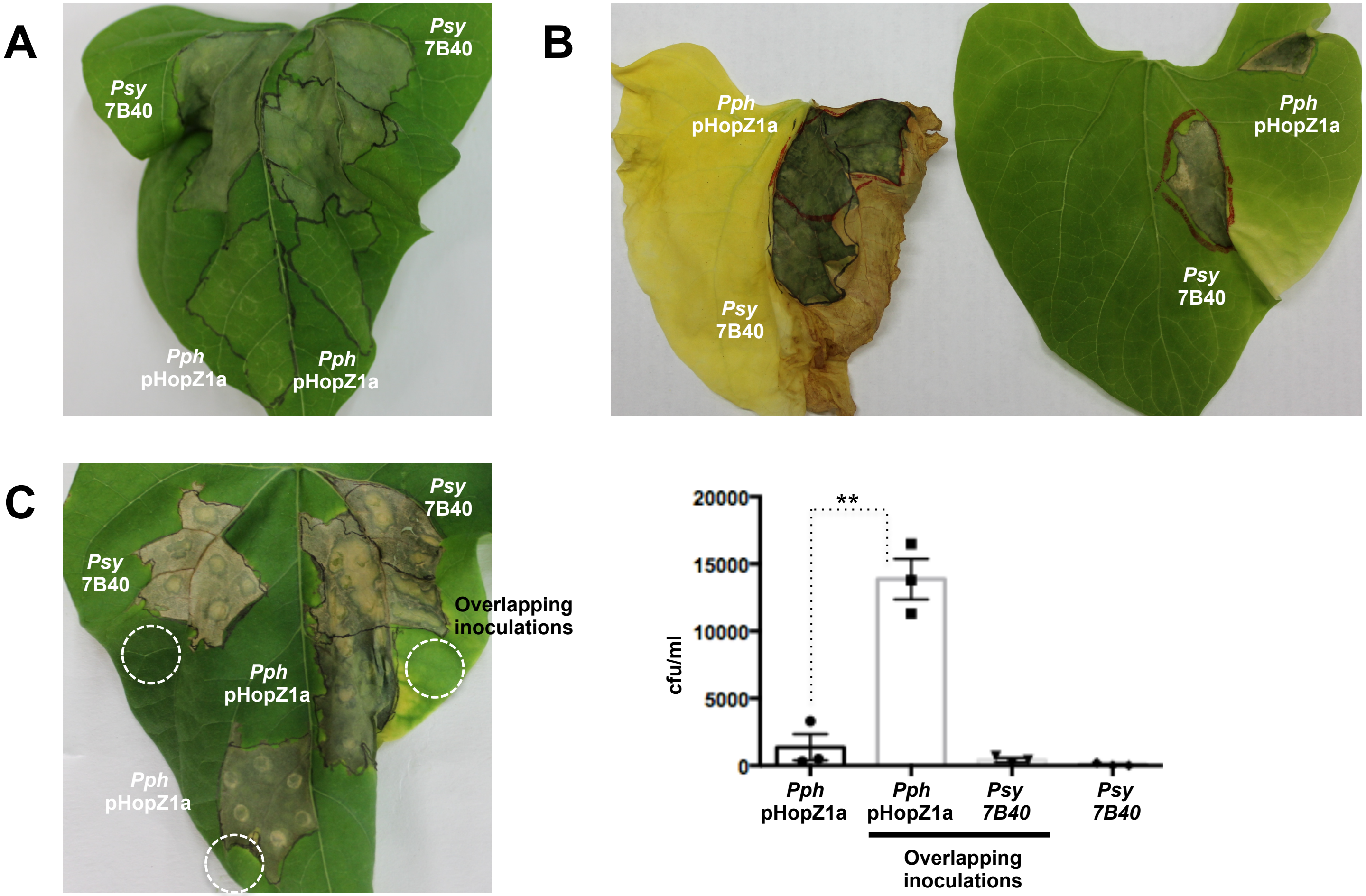

Fig. 2S Psy 7B40 suppresses *in trans* immunity triggered by HopZ1a when delivered from *Pph* 1448A pHopZ1a. Bean leaves were infiltrated with 10^7 cfu/ml of *Psy* 7B40 or *Pph* 1448A carrying pHopZ1a (pAME30) either separately or covering overlapping areas. (**A**) Images taken 24 hpi. The onset of HR is clearely seen in areas inoculated with *Psy* 7B40. HR in areas inoculated with *Pph* 1448A carrying pHopZ1a (pAME30) usually take between 24-36 hours to become apparent as visible necrosis. (**B**) When leaves are left longer (8-10 dpi), chlorosis spread from the areas co-inoculated with these two strains (left leaf) to the rest of the leave, which then becomes necrotic eventually falling off. This is in clear contrast with leaves inoculated with these strains separatedly (right leaf), where symptoms remain mostly constrained to the necrosis associated to the onset of the HR. For co-inoculations, Pph 1448A carrying pHopZ1a (pAME30) was infiltrated first (top right) and *Psy* 7B40 (bottom right) was infiltrated 2 hours later. Each experiment included at least three replicated. Experiments were repeated at least twice with similar results. (**C**) Left image shows similar experiment indicating areas where sampling to determine bacterial cfu was carried out. Graphon the right shows results obtained from that sampling. Mean data from four replicates are shown from a representative experiment. Error bars correspond to SE. Asterisks indicate mean values are significantly different as established by a t-test ($P<0.01$).
