# Supplementary material for: Suppression of HopZ Effector-Triggered Plant Immunity in a Natural Pathosystem
Source: Front Plant Sci. 2018 Aug 14;9:977. doi: 10.3389/fpls.2018.00977 (PMC6103241; doi:10.3389/fpls.2018.00977)
Supplement: Supplementary file 6 [file Image_3.PDF]

Plasmid library to screen for a suppressor of HopZ1a-triggered immunity in the 7B40 genome

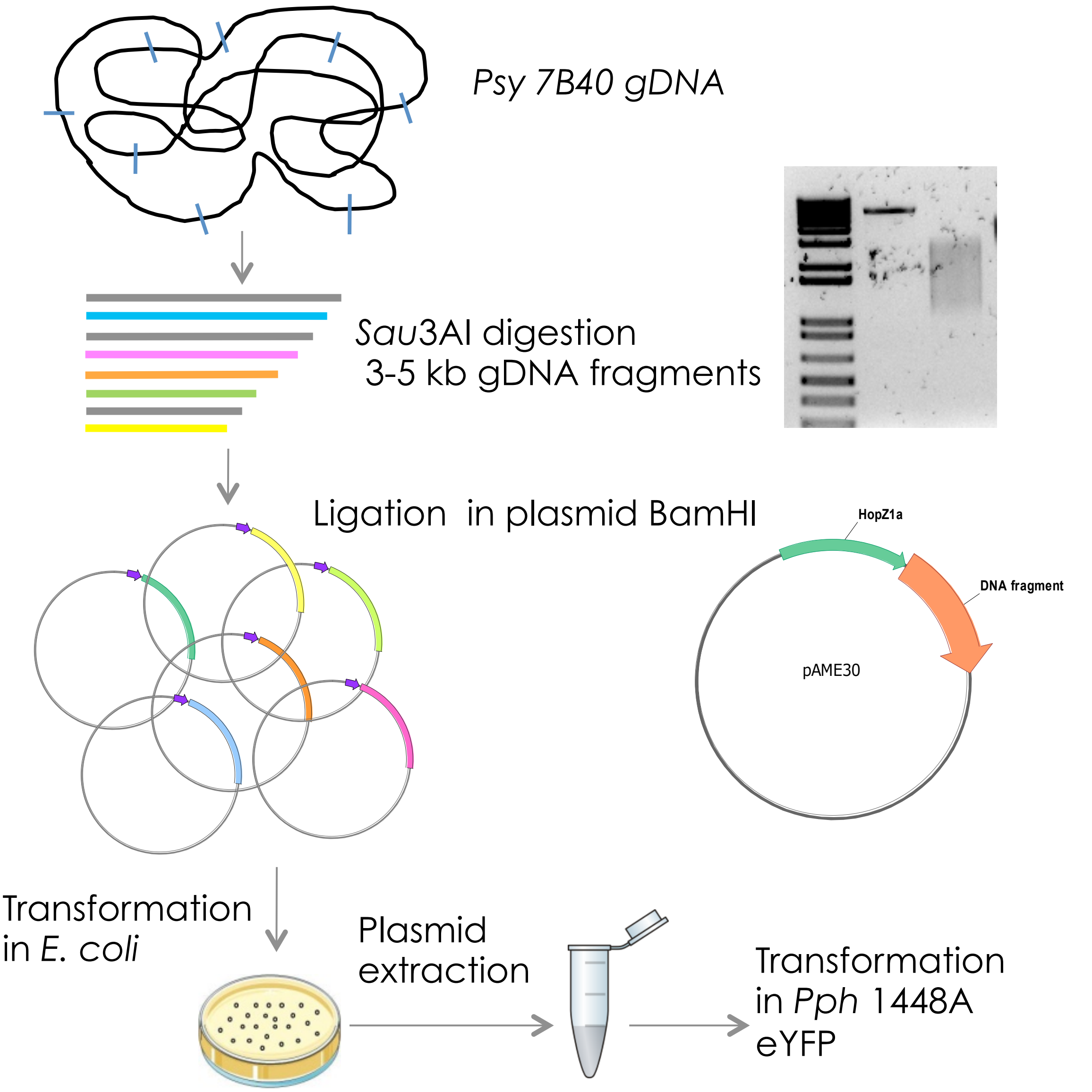

$$N = \frac{\ln(1-P)}{\ln(1-1/n)}$$

N = number of recombinant plasmids needed

P = probability of finding any sequence of your genome (95-99%)

n = genome size/insert average size

Clarke L and Carbon J (1976) Cell 9:91-99

Fig. 3S. Generating a plasmid library to screen for suppressors of HopZ1a-triggered immunity within the *Psy* 7B40 genome. *Psy* 7B40 genomic DNA was partially digested with Sau3AI to enrich in 3-5 kb fragments. These were cloned by ligation into the BamHI site of pAME30, located dowstream *PnptII::hopZ1a*. The resultant library was transformed into *E. coli* DH5α. An excess of 6,000 clones were pooled and subjected to plasmid extraction in order to transform the library into *Pph* 1448A eYFP. The resulting clones were use for the *in planta* screening
