# Supplementary material for: Suppression of HopZ Effector-Triggered Plant Immunity in a Natural Pathosystem
Source: Front Plant Sci. 2018 Aug 14;9:977. doi: 10.3389/fpls.2018.00977 (PMC6103241; doi:10.3389/fpls.2018.00977)
Supplement: Supplementary file 7 [file Image_4.PDF]

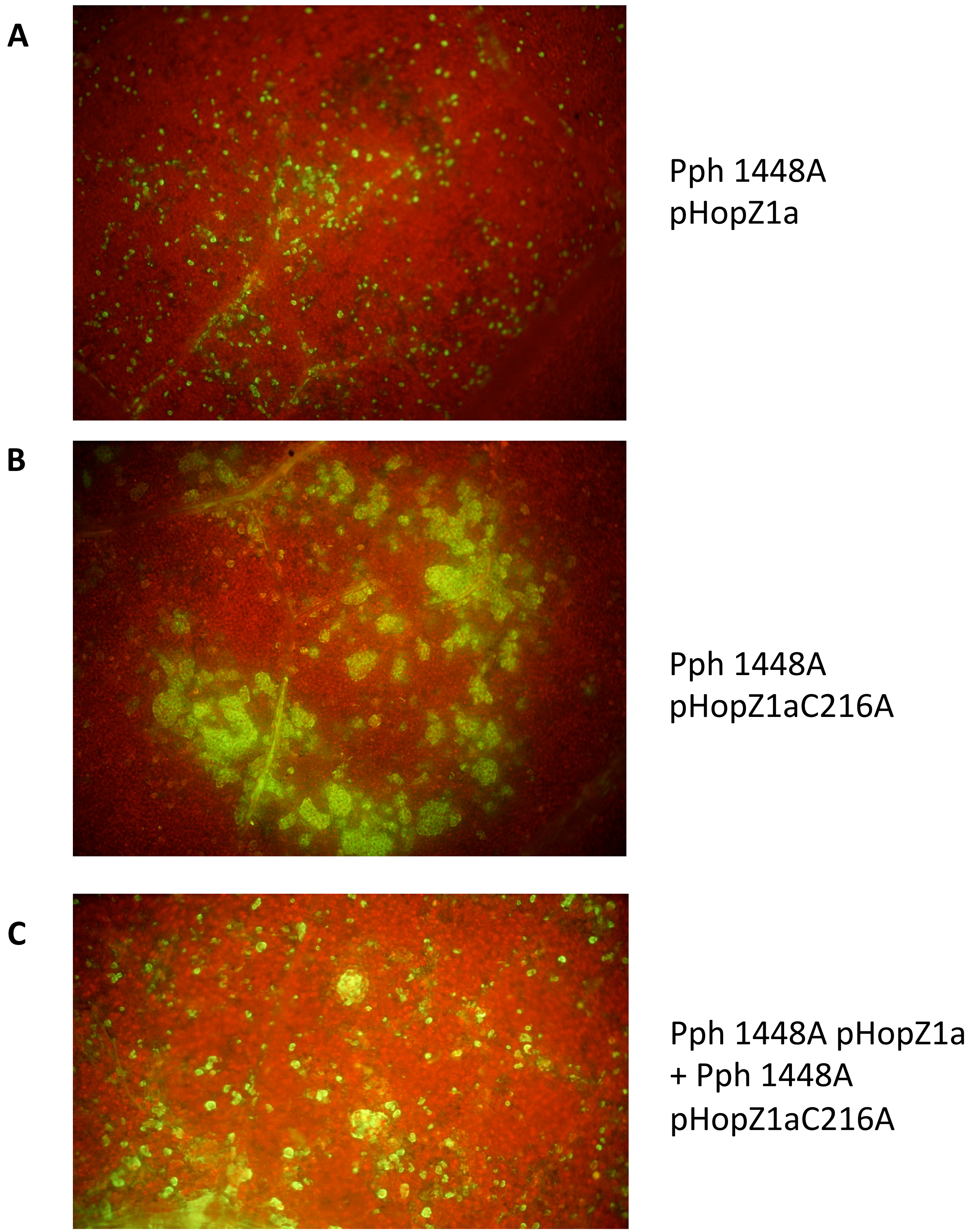

Fig. 4S Differences in microcolony development associated to expression of HopZ1a from *Pph* 1448A. Epifluorescence microscopy images show microcolonies of eGFP derivatives of *Pph* 1448A pHopZ1a (**A**, small microcolonies), *Pph* 1448A pHopZ1aC261A (B, large and confluent microcolonies) and a 1000:1 mix of *Pph* 1448A pHopZ1a and *Pph* 1448A pHopZ1aC261A (**C**, small and occassionally large microcolonies)
